# Molecular characterization and transcriptomic analysis of a novel polymycovirus in the fungus *Talaromyces amestolkiae*

**DOI:** 10.3389/fmicb.2022.1008409

**Published:** 2022-10-26

**Authors:** Li Teng, Sen Chen, Zuquan Hu, Jili Chen, Hongmei Liu, Tingting Zhang

**Affiliations:** ^1^Key Laboratory of Infectious Immune and Antibody Engineering of Guizhou Province, Engineering Research Center of Cellular Immunotherapy of Guizhou Province, School of Biology and Engineering/School of Basic Medical Sciences, Guizhou Medical University, Guiyang, China; ^2^Immune Cells and Antibody Engineering Research Center of Guizhou Province, Key Laboratory of Biology and Medical Engineering, Guizhou Medical University, Guiyang, China; ^3^Key Laboratory of Microbiology and Parasitology of Education Department of Guizhou, School of Basic Medical Science, Guizhou Medical University, Guiyang, China

**Keywords:** *Talaromyces amestolkiae*, genome, Polymycovirus, dsRNA virus, transcriptomic analysis

## Abstract

*Talaromyces amestolkiae* is an important fungal species owing to its ubiquity in soils, plants, air, and food. In this study, we identified a novel six-segmented polymycovirus, *Talaromyces amestolkiae* polymycovirus 1 (TaPmV-1). Each of the double-stranded (ds) RNA segments of TaPmV-1 contained a single open reading frame, and the proteins encoded by dsRNA1, dsRNA2, dsRNA3, and dsRNA 5 shared significant amino acid identities of 56, 40, 47, and 43%, respectively, with the corresponding proteins of *Aspergillus fumigatus* polymycovirus-1(AfuPmV-1). DsRNA1, dsRNA3, and dsRNA5 of TaPmV-1 encoded an RNA-dependent RNA polymerase (RdRp), a viral methyltransferase, and a PAS-rich protein, respectively. The functions of the proteins encoded by dsRNA2, dsRNA4, and dsRNA6 have not been elucidated. Comparison of the virus-infected strain LSH3 with virus-cured strain LSHVF revealed that infection with TaPmV-l may reduce the production of red pigments and induce the clustering of fungal sclerotia. Furthermore, transcriptomic analyses demonstrated that infection with TaPmV-l downregulated the expression of transcripts related to metabolism, and may correlate with the reduced production of red pigments and clustering of sclerotia in *T. amestolkiae*. These results of this study provide novel insights into the mechanism of fungal gene regulation by polymycovirus infections at the transcriptome level, and this study is the first to report a novel polymycovirus of *T. amestolkiae*.

## Introduction

The *Polymycovirus* genus, established in 2020, belongs to the *Polymycoviridae* family and is known to infect fungi (Kanhayuwa et al., 2015; Kotta-Loizou and Coutts, 2017; Mu et al., 2017; Mahillon et al., 2019; Nerva et al., 2019a; Gao et al., 2021; Kang et al., 2021; Kotta-Loizou et al., 2022; Ma et al., 2022). Polymycoviruses have multi-segmented and non-conventionally encapsulated double-stranded (ds) RNA genomes, with four to eight dsRNA segments (Kanhayuwa et al., 2015; Chiapello et al., 2020; García et al., 2020; Ma et al., 2022). Each of the segments contains an open reading frame (ORF) flanked by 5′- and 3′-untranslated regions (UTRs), that are highly conserved. In addition, all known polymycoviruses share four conserved genomic segments that encode an RNA-dependent RNA polymerase (RdRp), a hypothetical protein of unknown function (containing a transmembrane domain and a zinc-finger motif), putative methyltransferase (MTR), and a potential genome-associated protein (proline-alanine-serine-rich protein; PASrp; Kotta-Loizou et al., 2022). A recent study discovered a closely related single-stranded (ss) RNA virus with 11 genomic segments, named Hadaka virus 1 (Sato et al., 2020b).

Previous studies have demonstrated the majority of mycoviruses have no obvious effects on their hosts. However, previous studies have demonstrated that infection with polymycoviruses alters various features of hosts (Kanhayuwa et al., 2015; Zhai et al., 2016; Jia et al., 2017; Kotta-Loizou and Coutts, 2017; Niu et al., 2018; Takahashi-Nakaguchi et al., 2020; Filippou et al., 2021; Patil et al., 2021). For instance, it has been reported that infection with *Aspergillus fumigatus* polymycovirus 1 (AfuPmV-1) and *Botryoshaeria dothidea* polymycovirus 1(BdRV1) alters host virulence (Kanhayuwa et al., 2015; Zhai et al., 2016; Takahashi-Nakaguchi et al., 2020), which indicates that polymycoviruses can be exploited as potential biocontrol agents of disease-causing fungi. However, the majority of previous studies on polymycovirus-host fungus interactions are limited to comparisons of the biological characteristics of virus-infected and virus-cured strains.

Transcriptomic analysis is a powerful tool for elucidating the molecular interactions between viruses and hosts. Transcriptomic analysis aids the identification of biological processes or crucial host proteins that mediate virus-host interactions at the molecular level (Wang et al., 2016; Shimizu et al., 2018; Cao et al., 2019; Lee et al., 2021; Zhang et al., 2022). Transcriptomic technologies, especially RNA-seq, have widespread application in elucidating bacteriophage-host interactions (Jian et al., 2016; Mojardín and Salas, 2016; Morimoto et al., 2018; Yang et al., 2019; Zhong et al., 2020; Brandão et al., 2021; Zhang et al., 2022). However, few studies have investigated the transcriptional changes following fungal infections with mycoviruses, including *Cryphonectria parasitica, Botryosphaeria dothidea, Sclerotinia sclerotiorum,* and *Fusarium graminearum* (Wang et al., 2016, 2018; Ding et al., 2019; Chun et al., 2020; Qu et al., 2021). To date, several mycovirus-fungal interactions remain to be investigated using transcriptomic technologies.

The fungus *Talaromyces amestolkiae* has a widespread distribution, and has been cultured from the air of indoor spaces, lung tissues of patients with cystic fibrosis, soil, and manure (Yilmaz et al., 2012; Villanueva-Lozano et al., 2017). The sexual cycle of *T. amestolkiae* has been described in a previous study, under specific laboratory conditions(Yilmaz et al., 2016), and it has been reported that the production of a red diffusible pigment by *T. amestolkiae* is a crucial feature of this fungus (Yilmaz et al., 2012). To the best of our knowledge, there are no reports of mycoviral infections in *T. amestolkiae*.

In this study, we report the molecular characterization of a novel six-segmented polymycovirus, *Talaromyces amestolkiae* polymycovirus 1 (TaPmV-1), which infects the strain LH3 of *T. amestolkiae*. The findings revealed that TaPmV-1 shares significant amino acid similarity with AfuTmV-1, but has two more dsRNA segments. Additionally, infection with TaPmV-1 may reduce the production of red pigments and induce the clustering of fungal sclerotia. Finally, transcriptomic analysis was performed using RNA-seq, and the transcription profiles of the virus-infected and virus-cured strains were compared. The global changes in the transcriptional expression of TaPmV-1-infected *T. amestolkiae* were determined by transcriptomic analysis, which would aid in elucidating the molecular interactions between polymycoviruses and their fungal hosts in future studies.

## Materials and methods

### Fungal strains and culture conditions

TaPmV-1 was obtained from the strain LSH3 of a filamentous fungus, which was collected from a sample of sour soup in Guizhou Province, China. The strain LSH3 was identified by sequencing the amplified-internal transcribed spacer (ITS) region with polymerase chain reaction (PCR) using the primers enlisted in Supplementary Table S1. The results of BLASTn search revealed that the ITS sequence of the strain LSH3 was most similar to that of *T*. *amestolkiae* (data not shown). The strain LSH3 was cultured on potato dextrose agar (PDA) or potato dextrose broth (PDB) at 28°C.

### Extraction and purification of dsRNA

The fungal strain was cultured and harvested on cellophane membranes overlaid on the surface of PDA plates for 7 days. The mycelia were homogenized in liquid nitrogen, and the dsRNA was isolated using *CF*-11 cellulose columns (Sigma, St. Louis, MO, United States) as previously described (Peyambari and Roossinck, 2018). The prepared dsRNA was subsequently digested with S1 nuclease, ribonuclease A, and RNase-free DNase I (Takara, Dalian, China). The extracted dsRNA was analyzed by electrophoresis using 1% (w/v) agarose gels.

### cDNA cloning, sequencing, and sequence analysis

The purified dsRNA was used as the template for determining the complete dsRNA sequence as previously described (Lambden et al., 1992; Xie et al., 2006). The complete sequences thus obtained were analyzed using the BLAST algorithm of NCBI for the identification of homologous sequences. The potential ORFs were identified and translated using the DNAMAN software, with default parameters. The stem-loop structures of the terminal sequences of the viral RNAs were predicted using the RNA folding tool in the NUPACK webserver,^1^ with default settings. Multiple sequence alignments of the nucleotide and protein sequences were generated using the CLUSTAL X program version 2.0 (Larkin et al., 2007). Phylogenetic trees were constructed using the Maximum Likelihood (ML) method and JTT matrix-based model in MEGA11 (Jones et al., 1992; Tamura et al., 2021). The percentage of trees in which the associated taxa were clustered together is depicted beside the branches. For the heuristic search, the initial tree(s) were obtained by applying the Neighbor-Joining and BioNJ algorithms to a matrix of pairwise distances estimated using the JTT model, and the topology with superior log likelihood value was selected.

### Northern blotting

The purified dsRNA was separated by electrophoresis using 0.8% agarose gels under denaturing conditions. The dsRNA was transferred to a Hybond-N+ (Amersham GE) nylon membrane using the capillary-transfer method. Northern blotting was performed using DIG High Prime DNA Labeling and Detection Starter Kit II (Roche Diagnostics, Germany), according to the manufacturer’s instructions. The results were visualized using a Hamamatsu Photonics real-time image processor (Argus-50 model).

### Preparation of virus-cured *Talaromyces amestolkiae* strain

In order to eliminate the mycoviruses from the infected strain LSH3 of *T. amestolkiae*, the fungal strain was treated using the protoplast preparation and regeneration approach. Briefly, the fungal mycelia were harvested and washed thrice with 30 ml of sterile ddH_2_O and 10 ml of NaCl solution. The mycelia were added to freshly prepared 10 mg/ml snailase (Sigma-Aldrich, St. Louis, MO, United States) and NaCl, and subsequently incubated for 3 h at 30°C with gentle shaking (80 rpm). The protoplasts were then filtered through a 25 μM nylon mesh filter (Suzhou Sihong Filteration Solution Co., Ltd.) with 2 ml of STC solution (1.2 M Sorbitol, 10 mM Tris–HCl pH7.5, and 50 mM CaCl_2_) and centrifuged at 5000 rpm for 5 min at 4°C. Following centrifugation, the protoplasts were carefully removed and 5 ml of STC solution was added. The protoplast pellets (1 × 10^7^ cells/mL) were then pooled with 5 ml of STC solution and the centrifugation was repeated at 5000 rpm for 5 min at 4°C. The final protoplast pellet was re-suspended in 500 μl of STC solution. The absence of TaPmV-1 in the strain LSH3VF was confirmed by reverse transcription (RT)-PCR amplification using specific primers (Supplementary Table S1).

### Effects of TaPmV-1 infection on colony morphology, mycelial growth, and biomass of *Talaromyces amestolkiae*

The strains LSH3 and LSH3VF were grown on PDA plates for 7 days at 28°C, following which 7 mm agar plugs were removed from the actively growing margin and placed onto the middle of a PDA plate, and the growth was observed for up to 53 days. The morphology of the fungal colonies was additionally observed on days 7 and 53 of incubation. All the experiments were performed in triplicate for each fungal strain. Statistical analysis was performed using GraphPad Prism 8.0.1.

In order to assess biomass production, equal numbers of spores (3 × 10^5^ conidia/mL) of the strains LSH3 and LSH3VF were inoculated in 250 ml flasks containing 100 ml of PDB and incubated at 28°C on a rotary shaker at 200 rpm for 5 days. The mycelia were harvested and weighed for determining biomass production. All the experiments were performed in triplicate.

### Library preparation for transcriptome sequencing

The total RNA was extracted from the mycelia at 7 days for preparing the RNA samples. Briefly, the mRNA was purified from the total RNA using poly-T oligo-attached magnetic beads. The mRNA was subsequently segmented into small fragments using divalent cations under elevated temperatures using the First Strand Synthesis Reaction Buffer (5X). First-strand cDNA synthesis was performed using a random hexamer primer and M-MuLV Reverse Transcriptase, following which RNase H was used for degrading the RNA. Second-strand cDNA synthesis was subsequently performed using DNA Polymerase I and dNTP. The remaining overhangs were converted into blunt ends using exonucleases or polymerases. Following adenylation at the 3′ ends of the DNA fragments, adaptors with hairpin loop structures were ligated to the 3′ ends for hybridization. The fragments in the library were purified using an AMPure XP system (Beckman Coulter, Beverly, United States) for the preferential selection of cDNA fragments that were 370–420 bp in length. Following PCR amplification, the PCR products were purified using AMPure XP beads to obtain the final cDNA library.

The quality of the library was validated by subsequent tests. Following construction, the library was initially quantified using a Qubit2.0 fluorometer. The library was then diluted to a concentration of 1.5 ng/μL, and the insert size of the library was detected using an Agilent 2100 Bioanalyzer. After validating the insert size, the effective concentration of the library (>2 nM) was accurately quantified using RT-qPCR for assessing the quality of the library.

### Analysis of differentially expressed genes (DEGs) from RNA-seq data

The image data measured by the high-throughput sequencer were converted into sequence data (reads) by CASAVA base recognition analysis. The raw data (raw reads) in fastq format were first processed using the fastp software (Chen et al., 2018), and the clean data (clean reads) were obtained by removing the reads containing adapter sequences, reads with N bases, and low-quality reads. In addition, we used Bowtie2 software for aligning the post-quality control sequencing data with the ribosomal sequences from NCBI, Rfam, and other databases (Langmead and Salzberg, 2012; Langmead et al., 2019). The alignment results were counted and the sequences in the alignment were removed. The remaining clean reads were further used for assembly and calculation of gene abundance. An index of the reference genome of *T. amestolkiae* was constructed, and the paired-end clean reads were mapped to the reference genome using the HISAT2 program (de Eugenio et al., 2017; Kim et al., 2019). The mapped reads of each sample were assembled using SAMtools for obtaining the reconstructed transcript annotations (Li et al., 2009). The aligned fragments were imported into RSEQC and filtered using the default parameters (Wang et al., 2012). The expression levels of the transcripts were next determined using the RSEM software (Li and Dewey, 2011). The read counts of the transcripts were obtained, and converted into the read counts for the genes using the tximort package and Fragments Per Kilobase Million (FPKM), and the values of Transcripts Per Kilobase Million (TPM) were calculated (Soneson et al., 2015). The differential expression between the two different conditions/groups (two biological replicates per condition) was analyzed using the DESeq2 package (version 1.20.0) in R. The significantly different expression profiles were determined using padj<=0.05 and|log2(foldchange)| > =1 as the threshold values. The RNA-seq reads were trimmed using the Trim Galore script with default parameters for removing the Nextera adapter sequences.

### GO and KEGG enrichment analysis of DEGs

GO enrichment analysis of the DEGs was performed the clusterProfiler package in R (version 3.8.1), in which the bias in gene lengths was corrected. GO terms with corrected *p*-values less than 0.05 were considered to be significantly enriched. KEGG is a database resource for understanding high-level functions and utilities of biological systems, including cells, organisms, and the ecosystem, based on molecular information, especially large-scale molecular datasets generated by genome sequencing and other high-throughput experimental technologies.^2^ The clusterProfiler package in R (version 3.8.1) was also used for determining the statistically enriched KEGG pathways in the DEGs.

### Real-time -quantitative RT-PCR (RT-qPCR) analysis

The total RNA was isolated using an RNA extraction kit for fungi (OMEGA, United States). The cDNA was synthesized using the EasyScript^®^ One-Step gDNA Removal and cDNA Synthesis SuperMix, according to the manufacturer’s protocol. The concentrations of the RNA and DNA were determined and analyzed using a NanoDrop 2000 spectrophotometer (Thermo Scientific™) and agarose gel electrophoresis.

RNA extraction was performed as previously described. The cDNA was synthesized by qPCR using a Hifair^®^ II 1st Strand cDNA Synthesis SuperMix for qPCR kit. Real-time RT-qPCR was performed using the Hieff qPCR SYBR^®^ Green Master Mix with a QuantStudio™ 3 system (Thermo Scientific™). The volume of the PCR was 10 μl and the reaction mixture consisted of 5 μl of premix enzyme, 10 μM of each primer, and 1 μl cDNA. The primers for the target genes were designed using SnapGene version 4.1.18, and are enlisted in Supplementary Table S1. The results of RT-qPCR were normalized to those of the 16 s rRNA gene, which was used as the reference. PCR was performed under the following conditions: initial denaturation at 95°C for 5 min followed by 40 cycles of denaturation at 95°C for 10 s, extension at 55°C for 20 s, and annealing at 72°C for 30 s. Three independent experiments were performed, and the relative gene expression levels were analyzed using the 2^−ΔΔCT^ method.

## Results

### Molecular characterization of TaPmV-1 in the strain LSH3 of *Talaromyces amestolkiae*

**Figure 1:**
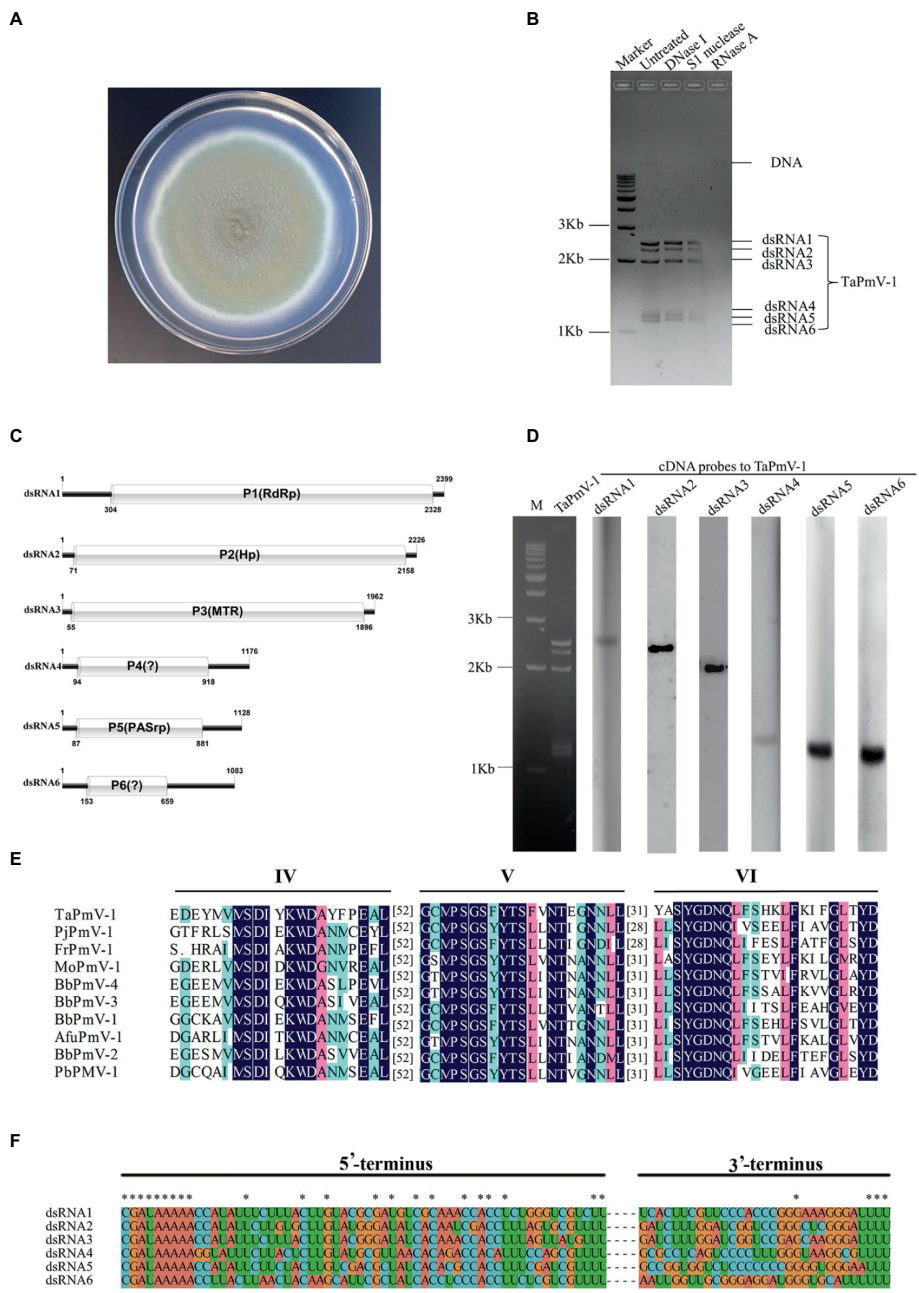
Genomic characteristics and organization of TaPmV-1. **(A)** Colony morphology of strain LSH3. **(B)** Electrophoretic profile obtained using 1% agarose gels, of dsRNA preparations extracted from the strain LSH3 following digestion with DNase I, S1 nuclease, or RNase A, and the untreated group. **(C)** Organization of dsRNAs 1–6 of TaPmV-1. **(D)** Detection of dsRNAs 1–6 of TaPmV-1 by northern blotting. Lane 1 depicts the dsRNA profile of TaPmV-1, and lanes 2, 3, 4, 5, 6, and 7 represent the individual transfers hybridized with specific probes for dsRNA1, dsRNA2, dsRNA3, dsRNA4, dsRNA5, and dsRNA6, respectively. **(E)** Comparison of the conserved motifs of the RdRp protein encoded by TaPmV-1 with those of other polymycoviruses. **(F)** Conserved sequences at the 5′- and 3′-termini of dsRNAs 1–6 of TaPmV-1.

*Talaromyces amestolkiae* strain LSH3 was isolated and identified using the ITS-PCR sequencing approach (refer Materials and Methods). The colony morphology of strain LSH3 is depicted in Figure 1A. Six dsRNA segments with sizes of 1–3 Kb were detected in the host strain LSH3 (Figure 1B). The complete sequences of the six dsRNA segments, namely, dsRNA1, dsRNA2, dsRNA3, dsRNA4, dsRNA5, and dsRNA6 were 2,399, 2,226, 1962, 1,176, 1,128, and 1,083 bp, respectively. The organization of the six dsRNA segments (dsRNA1-dsRNA6) is depicted in Figure 1C. The corresponding RNA segments were determined by northern hybridization using cDNA probes specific for the six dsRNA segments (Figure 1D). Each of the segments contained a single ORF which encoded proteins (P1-P6) comprising 673, 694, 612, 273, 263, and 177 amino acids, with molecular weights of 73.9, 75.0, 64.9, 29.1, 28.1, and 18.5 kDa, respectively.

The results of BLASTX search revealed that proteins P1, P2, P3, and P5 shared significant amino acid identities of 55.86, 39.54, 46.59, and 43.35%, respectively, with the corresponding proteins of AfuPmV-1, which belongs to the *Polymycovirus* genus (Kanhayuwa et al., 2015; Zoll et al., 2018). However, proteins P4 and P6 did not exhibit similarity to any known protein. These results demonstrated that the six dsRNA segments (dsRNA1-dsRNA6) represent a putative *Polymycovirus*, and the name *T. amestolkiae* polymycovirus 1 (TaPmV-1) was proposed for this virus. The complete genome of TaPmV-1 has been deposited in GenBank under accession numbers OP096450-OP096455.

Analysis of sequence homology revealed that the protein encoded by dsRNA1 of TaPmV-1 was most closely related to the RdRp protein encoded by Phaeoacremonium minimum tetramycovirus 1 (PmTMV1), and the proteins shared 56.33% amino acid sequence identity (accession number: QDB74985.1, e-value = 0.00) (Nerva et al., 2019b). The RdRp protein of TaPmV-1 comprised conserved domains IV, V, and VI of the picorna-like RdRp family (RdRP_1, PF00680), and the GDD motif was replaced by the GDNQ motif (Figure 1E). This is a typical feature that is observed in the RdRp proteins of other polymycoviruses as well (Kotta-Loizou and Coutts, 2017; Niu et al., 2018).

DsRNA2 of TaPmV-1 encoded a hypothetical protein containing a cysteine-rich zinc finger-like motif (Supplementary Figure S1), which shared low sequence similarity with the hypothetical protein encoded by dsRNA2 of AfuPmV-1 (accession number: CDP74619; e-value = 3e-161; and 39.54% identity). The hypothetical protein is assumed to act as a scaffold during viral replication, and function as a chaperone for dsRNAs in other polymycoviruses (Kotta-Loizou and Coutts, 2017).

DsRNA3 of TaPmV-1 encoded a protein that shared the highest identity of 48.44% with an MTR encoded by dsRNA3 of PmTMV1 (accession number: QDB74987.1; e-value = 1e-118), followed by 46.59% identity with an MTR encoded by dsRNA3 of AfuPmV-1 (accession number: BBU42082.1; e-value = 2e-136). Moreover, we observed that the N-terminal region (residues 133–235) of the protein encoded by dsRNA3 of TaPmV-1 contained a conserved S-adenosylmethionine-dependent MTR catalytic motif (pfam13649 and e-value = 1.36e-07; Supplementary Figure S2). The MTR domain that RNA-capping is conserved in the alphavirus-like superfamily and present in some picorna-like mycoviruses (Howitt et al., 2001; Roossinck et al., 2011). The protein possibly catalyzes the addition of a cap structure at the 5′ -terminus of the viral RNA (Kotta-Loizou and Coutts, 2017). In addition, an essential lysine residue was identified MTR domain of TaPmV-1 and other polymycoviruses in this study (Supplementary Figure S3).

DsRNA5 of TaPmV-1 encoded a PASrp, in which proline, alanine, and serine residues comprised 5.94, 7.85, and 7.98%, respectively, of the 261 residues. This protein shared 46.56% sequence identity with a PASrp encoded by dsRNA4 of PmTMV1 (accession number: QDB74988.1; e-value = 1e-66). PASrp is assumed to act as viral capsids for protecting the genomic RNA (Sato et al., 2020a).

DsRNA4 and dsRNA6 of TaPmV-1 contain one ORF which encodes proteins P4 and P6, respectively (Figure 1C). However, the proteins encoded by dsRNA4 and dsRNA6 were not homologous to any known protein; therefore, their functions in the viral life cycle remain to be elucidated.

The 5′ -UTRs of the coding strands in dsRNA1, dsRNA2, dsRNA3, dsRNA4, dsRNA4, dsRNA5, and dsRNA6 of TaPmV-1 were 303, 70, 54, 93, 86, and 152 bp in length, respectively, while the 3’-UTRs were 73, 70, 68, 260, 249, and 426 bp in length, respectively. The 5′- and 3′-UTRs of the six dsRNA segments of TaPmV-1 were highly conserved (Figure 1F). The 5’-UTRs of the coding strands of TaPmV-1 contained a conserved CGATAAAAA sequence, while the 3’-UTRs contained a conserved TTT sequence. The 5′- and 3′-UTRs were predicted to fold into stable stem-loop structures (Supplementary Figure S4).

### Phylogenetic analysis of TaPmV-1

**Figure 2:**
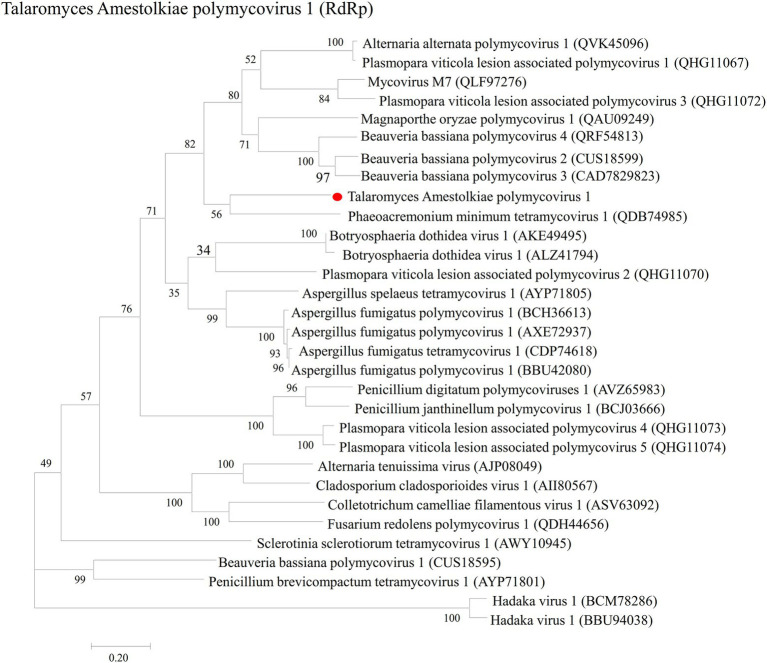
Phylogenetic analyses of TaPmV-1 and selected polymycoviruses. Phylogenetic analyses of TaPmV-l based on the sequences of RdRp.

In order to analyze the phylogenetic relationships of TaPmV-1, a phylogenetic tree was constructed based on the amino acid sequences of the RdRp protein of TaPmV-1, and selected proteins encoded by other dsRNA viruses using the ML method (Supplementary Table S2). The results demonstrated that the RdRp protein of TaPmV-1 was most closely related to the RdRp proteins of other polymycoviruses (Figure 2).

In addition, the phylogenetic relationship of TaPmV-1 with the *Polymycovirus* genus was additionally analyzed based on the amino acid sequences of MTP and PASrp using the ML method (Supplementary Table S2). The phylogenetic tree constructed using the MTP sequences suggested that TaPmV-1 was most closely related to PmTMV1, while the phylogenetic tree constructed using the PASrp sequences revealed that TaPmV-1 was most closely related to PmTMV1, BbPmV3, and MoPmV1 (Supplementary Figures S5, S6).

Based on these results of phylogenetic analyses, we propose that the TaPmV-1 is a novel virus of the *Polymycoviridae* family.

### Effects of TaPmV-1 infection on colony morphology, mycelial growth, and biomass of *Talaromyces amestolkiae*

**Figure 3:**
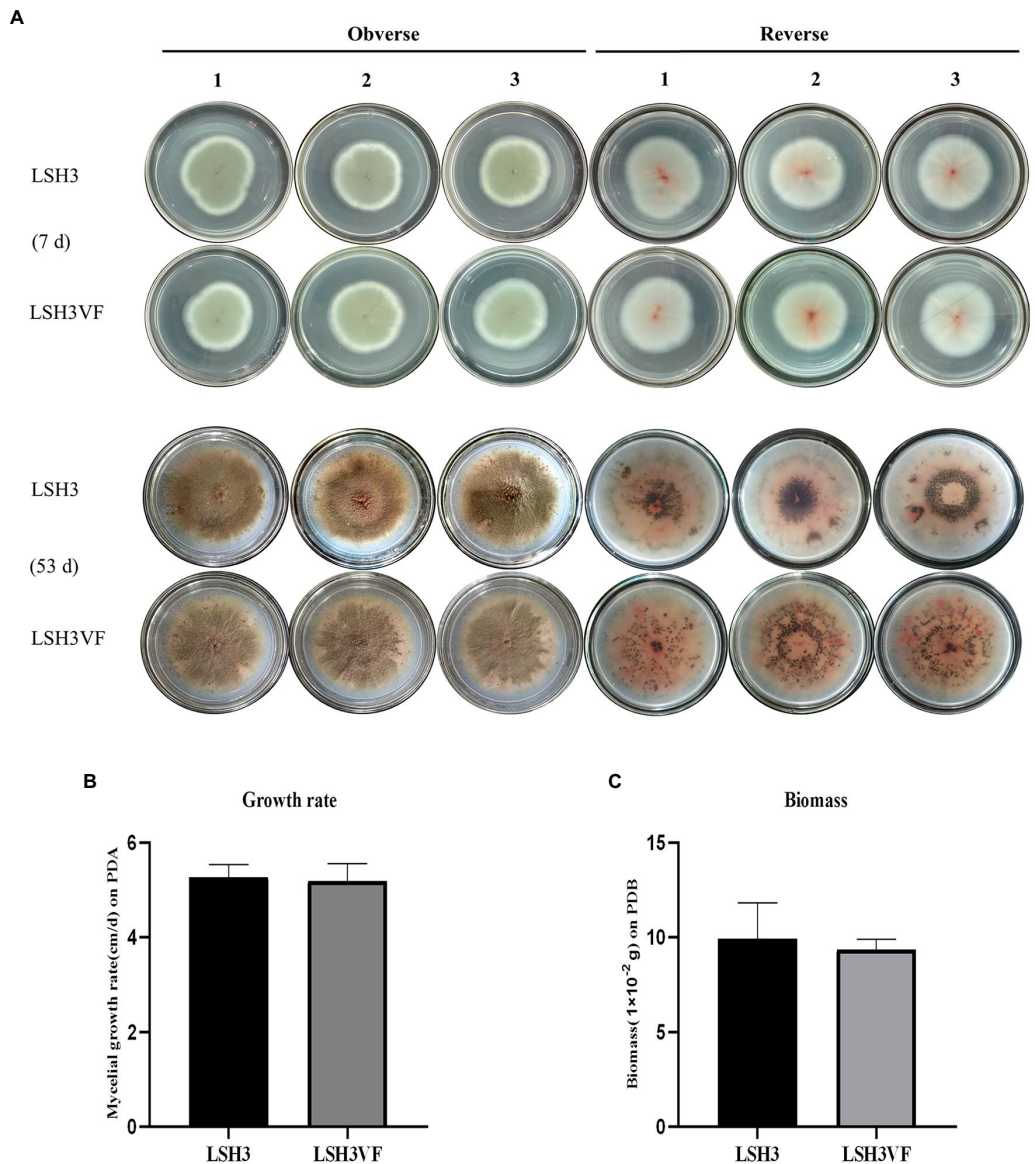
Biological effects of TaPmV-l on the virus-infected strain LSH3 and the virus-cured strain LSH3VF. **(A)** Comparison of the colony morphology formed by the virus-infected strain LSH3 and the virus-cured strain LSH3VF at 7 and 53 days, respectively. **(B)** Comparison of the growth of the virus-infected strain LSH3 and the virus-cured strain LSH3VF. **(C)** Comparison of biomass production in the virus-infected strain LSH3 and the virus-cured strain LSH3VF.

In order to assess the effects of mycoviruses on the host fungus, a virus-cured strain LSH3VF was obtained from the parental strain LSH3 based on the protoplast preparation and regeneration approach. The virus-cured strain LSH3VF was confirmed by RT-PCR amplification, using specific primers (Supplementary Figure S7; Supplementary Table S1). Previous studies have demonstrated that the *T. amestolkiae* fungus can produce black sclerotia (Yilmaz et al., 2016). The strains LSH3 and LSH3VF were therefore incubated for up to 53 days, and colony morphology was observed at days 7 and 53 (Figure 3A). The results demonstrated that the strains LSH3 and LSH3VF had little red pigment at 7 days of incubation, but did not produce black sclerotia, and exhibited no differences (Figure 3A). However, observation of the strains LSH3 and LSH3VF at 53 days of incubation revealed that both strains produced large quantities of red pigment and developed black sclerotia, and the production of red pigments and the distribution of sclerotia were different between the strain LSH3 and LSH3VF (Figure 3A). Additionally, there were no significant differences in mycelial growth and biomass accumulation between the strains LSH3 and LSH3VF, as depicted in Figures 3B,C. Phenotypic changes are known to be linked to differential gene expression; therefore, the results of this study indicated that further transcriptomic analyses are necessary for obtaining detailed insights into the effect of TaPmV-l on the host fungus.

### Transcriptional changes in *Talaromyces amestolkiae* infected with TaPmV-1

**Figure 4:**
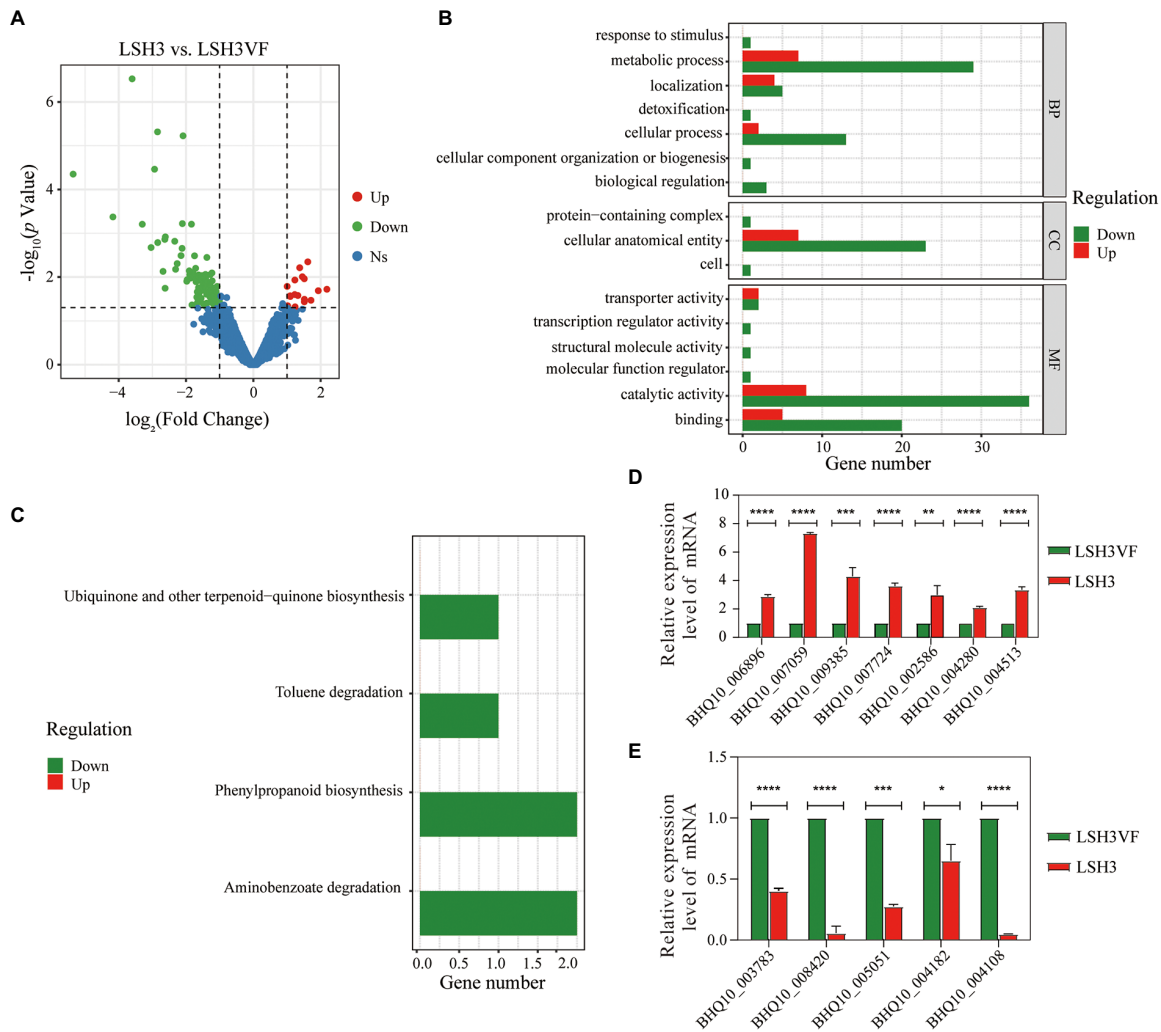
**(A)** Comparison of the profiles of the DEGs of the virus-infected strain with those of uninfected *T. amestolkiae*. **(B,C)** The clusterProfiler package in R was used for GO enrichment analysis, and KEGG enrichment analysis of the DEGs. **(D,E)** RT-qPCR analysis of the upregulated and downregulated DEGs.

In order to characterize the transcriptional changes following TaPmV-1 infection in *T. amestolkiae*, transcriptome sequencing (RNA-seq) analysis was performed for the virus-infected strain LSH3 and the virus-cured strain LSH3VF. The total RNA was extracted from the mycelia at 7 days of incubation for preparing the RNA samples. However, the mycelia at 53 days of incubation were not selected for preparing the RNA samples, as the mycelia were too old for total RNA extraction. A total of 9,915 gene transcripts were obtained. We identified 117 DEGs from the strain LSH3 (Figure 4A; Supplementary Table S3), and the expression of more than 85% of these DEGs was downregulated. In order to elucidate the functions of the DEGs that were regulated by TaPmV-1 infection, we determined the GO terms that were significantly enriched in the DEGs (Supplementary Table S4). The identified GO terms were classified into the biological process (BP), cellular component (CC), and molecular function (MF) categories. The results of GO analysis revealed that these DEGs were significantly enriched in cellular anatomical entity for CC, metabolic process for BP, and catalytic activity for MF (Figure 4B). The findings implied that the metabolic processes of the virus-infected strain LSH3 may undergo alterations following viral infections. Consistently, the results of KEGG enrichment analysis revealed that these DEGs were involved in multiple metabolic pathways, including ubiquinone and other terpenoid-quinone biosynthesis, phenylpropanoid biosynthesis, aminobenzoate degradation, and toluene degradation (Figure 4C; Supplementary Table S5). We additionally determined the expression of the DEGs that were related to metabolism by RT-qPCR (Supplementary Table S6). The results revealed that the expression of genes encoding sterol regulatory element-binding protein (BHQ10_002586), amino oxidase (BHQ10_009385), polyprenyl synthetase (BHQ10_006896), cytochrome P450 (BHQ10_004280), major receptor superfamily (BHQ10_007724), and sugar isomerase (BHQ10_007059) was upregulated, while the expression of genes encoding fungal hydrophobin (BHQ10_008420), AMP-binding enzyme (BHQ10_005051), ATP-binging cassettes (BHQ10_004108), UbiA family of prenyltransferases (BHQ10_003783), and flavoprotein (BHQ10_004182) was downregulated (Figures 4D,E). These findings were consistent with the results of RNA-seq analysis. A previous study reported that *T. amestolkiae* is a bioresource for the production of natural red colorants (de Oliveira et al., 2019). Despite alterations in the expression of these genes between the virus-infected strain LSH3 and the virus-cured strain LSHVF at 7 days of incubation, no significant phenotypic changes were observed in this study (Figure 3A). However, the production of red pigments and distribution of sclerotia differed significantly between the strain LSH3 and LSH3VF at 53 days of incubation (Figure 3A). These findings implied that these genes could play an important role in the differences in the production of red pigments and distribution of sclerotia. Significant phenotypic changes were not observed at 7 days of incubation as the strain LSH3 and LSH3VF produced little red pigment and did not produce black sclerotia at 7 days (Figure 3A). ATP-binding cassettes play important roles in secondary metabolism, and the UbiA family of prenyltransferases plays key roles in the production of biotin. Some studies have demonstrated that *T. amestolkiae* is able to degrade lignocellulose (Méndez-Líter et al., 2020, 2021), and the gene encoding sugar isomerase (BHQ10_007059) may be involved in lignocellulose degradation. These findings suggest that the virus-infected strain LSH3 may regulate the production of red pigments and lignocellulose degradation by affecting the related metabolic processes. Notably, we observed that the expression of genes related to pleiotropic drug resistance (BHQ10_004513 and BHQ10_002586) was upregulated following viral infections, indicating that the drug resistance of the virus-infected strain may also be affected following TaPmV-1 infections (Kanhayuwa et al., 2015; Figure 4D). We observed that the gene encoding the ATP-binging cassette (BHQ10_004108), which is associated with multidrug resistance, was also downregulated (Howitt et al., 2006; Figure 4E). Altogether, the results suggested that TaPmV-1 infection may affect the metabolic processes of biotin production and lignocellulose degradation, and drug resistance of the virus-infected strain LSH3. The transcriptome data obtained herein can provide important molecular genomic information for further functional characterization analysis of *T. amestolkiae* in response to polymycovirus infections.

## Discussion

*Talaromyces amestolkiae* is a common heterothallic *Penicillium*-like fungus. It has been isolated from the air of indoor spaces, sputum and lungs of patients with cystic fibrosis, wheat, soil, sculptures, and chicken-feed, among other sources (Yilmaz et al., 2012). To the best of our knowledge, there are no reports of mycoviral infections of *T. amestolkiae* to date.

In this study, a novel mycovirus, TaPmV-1, was identified from the strain LSH3 of *T. amestolkiae*. The genome of TaPmV-1 comprises six dsRNA segments, with each dsRNA segment consisting of a single ORF (Figure 1C). We determined that the P1, P2, P3, and P5 proteins of TaPmV-1 shared the highest amino acid sequence identities of 40–56% with the corresponding proteins of AfuPmV-1, which infects the human pathogenic fungus, *A. fumigatus* (Kanhayuwa et al., 2015). The P2 protein was rich in arginine repeats, which are considered to be endoplasmic reticulum (ER) retention signals and are normally found in transmembrane proteins and partake in viral replication (Kotta-Loizou and Coutts, 2017). The P5 protein was rich in PAS domains, which are frequently found in certain scaffold proteins and have been associated with protein–protein interactions (Spear et al., 2010). However, the P4 and P6 proteins encoded by dsRNA5 and dsRNA6 did not show similarities to any known proteins in the NCBI database. Further studies are therefore necessary for elucidating the functions of P5 and P6 proteins of TaPmV-1. The 5’-UTRs of the dsRNAs of TaPmV-1 were highly similar to the corresponding sequences of AfuPmV-1 and BdRV1 (Figure 1F). However, the 3’-UTRs of the dsRNAs of TaPmV-1 were not highly similar to the corresponding sequences of AfuPmV-1 and BdRV1. In most cases, the conserved sequences at the terminal ends of viral genomic RNAs play important roles in viral packaging (Wei et al., 2003).

Polymycovirus infections are associated with various alterations in hosts, including changes in pigmentation, sectoring, and decreased host growth and host virulence, as well as increased sporulation, host growth, host virulence, and improved sensitivity to antifungals (Kanhayuwa et al., 2015; Zhai et al., 2016; Kotta-Loizou and Coutts, 2017; Niu et al., 2018; Takahashi-Nakaguchi et al., 2020; Filippou et al., 2021; Patil et al., 2021). In this study, we therefore compared the colony morphology, growth rate, and biomass production of the virus-infected strain LSH3 with those of the virus-cured strain LSH3VF. These results demonstrated that TaPmV-l infections may reduce the production of red pigments and induce the clustering of fungal sclerotia (Figure 3A). Previous studies have demonstrated that infection with other polymycoviruses affects pigment production in the host (Kanhayuwa et al., 2015; Filippou et al., 2021). Further studies are therefore necessary for determining the factor responsible for these differences.

The measurement of mRNA expression patterns at the transcriptome level following infection with polymycoviruses in fungi is vital for understanding the mechanism underlying mycovirus-mediated alterations in pathogenic fungi. Previous studies have demonstrated that polymycovirus infections alter the phenotypes of virus-infected and isogenic virus-free strains (Kanhayuwa et al., 2015; Zhai et al., 2016; Kotta-Loizou and Coutts, 2017; Niu et al., 2018; Takahashi-Nakaguchi et al., 2020; Filippou et al., 2021; Patil et al., 2021). However, there are no reports on the alterations in transcript accumulation in polymycovirus-infected fungi. We therefore performed transcriptome sequencing (RNA-seq) analysis for identifying the alterations in transcriptional expression in *T. amestolkiae* following TaPmV-l infection. A total of 117 genes were found to be differentially expressed following TaPmV-l infection, and the expression of more than 85% of these DEGs was downregulated GO and KEGG analyses revealed that several of the identified DEGs were significantly enriched in metabolic pathways (Figures 4B,C). These results suggested that infection with TaPmV-l caused specific alterations in the transcriptome of *T. amestolkiae*, and the downregulation of the transcripts related to metabolism may correlate with the reduced production of red pigments and increased clustering of sclerotia. This study serves as an excellent example for studying host-virus interactions in future studies.

## Data availability statement

The datasets presented in this study can be found in online repositories. The names of the repository/repositories and accession number(s) can be found in the article/Supplementary material.

## Author contributions

TZ and HL conceived and designed the experiments. LT and SC performed the experiments. JC performed some of the experiments. ZH analyzed the data. TZ prepared the manuscript. All authors contributed to the article and approved the submitted version.

## Funding

This research was supported by the Guizhou Provincial Natural Science Foundation (grant number: ZK [2021]081), the National Natural Science Foundation of China (grant numbers: 32160668 and 12132006), and the Guizhou Provincial Natural Science Foundation for High-Level Innovative Talents and Teams (grant numbers: 2016–5676 and 2015–4021).

## Conflict of interest

The authors declare that the research was conducted in the absence of any commercial or financial relationships that could be construed as a potential conflict of interest.

## Publisher’s note

All claims expressed in this article are solely those of the authors and do not necessarily represent those of their affiliated organizations, or those of the publisher, the editors and the reviewers. Any product that may be evaluated in this article, or claim that may be made by its manufacturer, is not guaranteed or endorsed by the publisher.

## Footnotes

^1^ http://www.nupack.org/partition/new

^2^ http://www.genome.jp/kegg/
