# Supplementary material for: Molecular characterization and transcriptomic analysis of a novel polymycovirus in the fungus Talaromyces amestolkiae
Source: Front Microbiol. 2022 Oct 26;13:1008409. doi: 10.3389/fmicb.2022.1008409 (PMC9645161; doi:10.3389/fmicb.2022.1008409)
Supplement: Supplementary file 2 [file Table_1.DOCX]

**Table S1** The primer sequence used in this study.

| **Primer** | | **Sequence (5’-3’)** | **Description** |
| --- | --- | --- | --- |
| TaPmV-1-5mo-1 | CAACCAGAGTTAACGCAGCCACG | | **5' and 3' RACE** |
| TaPmV-1-5mo-2 | CATGGAGAGGACCAAGGAACGG | |  |
| TaPmV-1-3mo-1 | CTTAAGGTTGACGAGACGGAGGTC | |  |
| TaPmV-1-3mo-2 | GATCAGCATGCAGCGCACCTG | |  |
| TaPmV-2-5mo-1 | CACCAATTGATCCAAGGACAGGCC | |  |
| TaPmV-2-5mo-2 | GGAATTGAACCAGCAGGTAAGGGATGAC | |  |
| TaPmV-2-3mo-1 | CAAGCTCGGCGACTCCGATGC | |  |
| TaPmV-2-3mo-2 | CGCATATCGCATGCTCTCGAG | |  |
| TaPmV-3-5mo-1 | GAAGGGTAACAGAAGATCCAGAGGGAGTC | |  |
| TaPmV-3-5mo-2 | GGATGAGCCCGATTCACTAGGAG | |  |
| TaPmV-3-3mo-1 | CGGCGTCATCACTGAATTTAGGAGG | |  |
| TaPmV-3-3mo-2 | GGCGGGACGTATTAGGAATGACAAG | |  |
| TaPmV-4-5mo-1 | CTTCCCTAGATGCATAAGGGGGAAG | |  |
| TaPmV-4-5mo-2 | CCGGACGAGAAGGCAATAGAC | |  |
| TaPmV-4-3mo-1 | GTGTTAATGCGTATAGTCCTGGTGTG | |  |
| TaPmV-4-3mo-2 | GGACTGTCCTTCTCCAGTGATGC | |  |
| TaPmV-5-5mo-1 | GGTGATGATAGAAGAGAGCGACATAGTGAG | |  |
| TaPmV-5-5mo-2 | GGTCAGGCCCTTAAGCTTGGC | |  |
| TaPmV-5-3mo-1 | GTTTGCTGCGTTTAGTTGTCCTTATTTGG | |  |
| TaPmV-5-3mo-2 | GAGATGTTTGCTTTAGCCTCCAGTG | |  |
| TaPmV-6-5mo-1 | GAGGGCGTAGCCCTTGAAAGAAAC | |  |
| TaPmV-6-5mo-2 | GTCTGATCCCAGAAAACAGCCGGG | |  |
| TaPmV-6-3mo-1 | CTGCTGGTTCCTAAACCGAACCTTG | |  |
| TaPmV-6-3mo-2 | GTCCACCGCCTTAGTAGGAATGTCTG | |  |
| REV-PCR  REV-anchor | TCACTAAAGAATTCGATCGATC  GCATTGCATCATGATCGATCGAATTCTTTA  GTGAGGGTTAATTGCC  (5’ end phosphorylated oligonucleotide) | |  |
| Probe-dsRNA1F | CTTGTACGCGATGTCGCAAACC | | **Northern Blot** |
| Probe-dsRNA1R | CCAATAGTGGCCTGCTGTCG | |  |
| Probe-dsRNA2F | CACAATCGACCTTTAGTGCGTTTTCTG | |  |
| Probe-dsRNA2R | CGAGCGATGACAGAGCATCAAGC | |  |
| Probe-dsRNA3F | CTTGTACGGGATATCACAAACCACCTTTAG | |  |
| Probe-dsRNA3R | CTGACCGATGCACTTAGTCGC | |  |
| Probe-dsRNA4F | CTCTTGTATGCGTTAACACAGACCAC | |  |
| Probe-dsRNA4R | GCACACTAACATAAGAGTGGGCAC | |  |
| Probe-dsRNA5F | GTTCTGATAAGAAAGCACTCAAGGCCG | |  |
| Probe-dsRNA5R | GAATCTATCAAGCGACAGGAATCACACTG | |  |
| Probe-dsRNA6F | GTGTTGTACACGGCCGTGG | |  |
| Probe-dsRNA6R | CCCGCAACCAATTCGTGCC | |  |
| Probe-dsRNA1F | CTTGTACGCGATGTCGCAAACC | | **RT-PCR** |
| Probe-dsRNA1R | CCAATAGTGGCCTGCTGTCG | |  |
| ITS1 | TCCGTAGGTGAACCTGCGG | | **ITS-PCR** |
| ITS4 | TCCTCCGCTTATTGATATGC | |  |
| ITSF | CTCTGTGAACCCTGATGAAGATGGG | | **RT-qPCR** |
| ITSR | GATTCGATGATTCACGGAATTCTGCAATTC | |  |
| BHQ10_002586F | CTCCTTAACGCACAGCTCAAGC | |  |
| BHQ10_002586R | GTTGTGAGCAGCTTTGTTGACAACG | |  |
| BHQ10_009385F | CCATATATTGCGTATACCGCAGCAG | |  |
| BHQ10_009385R | CAGCAACTAACAGCGCAAGTTCC | |  |
| BHQ10_004513F | GGAGTCATCATCAGTGTATCGTGAGTTG | |  |
| BHQ10_004513R | GCTCTGTACAATGATAGTCACAGGCTC | |  |
| BHQ10_006896F | GGAACCATCTAAACCGACCTCGG | |  |
| BHQ10_006896R | GGCAAATTTGTGTACACGCAGAGG | |  |
| BHQ10_004280F | CACATCTCCGAGCGGAACTCTTAG | |  |
| BHQ10_004280R | GGCAGACTATTCAAGGACTGTGC | |  |
| BHQ10_007724F | GTGTTGGTGCTCAGTATGCAGATTG | |  |
| BHQ10_007724R | CCGAGGGAGTATGTTGGTGATTGATC | |  |
| BHQ10_007059F | GTCTCGCAACTCGTTCACACAATC | |  |
| BHQ10_007059R | GGAGTGTGTCAATAGATCGAATAACGCC | |  |
| BHQ10_008420F | GAAGTGCCAATGAGAAGGAAGTGAG | |  |
| BHQ10_008420R | CGACGTCCAACTGAGATATACGTG | |  |
| BHQ10_005051F | CTAATCTCTGCCACACCTGTAGCC | |  |
| BHQ10_005051R | CCGAAACTTTCATGCCAAGCTCC | |  |
| BHQ10_004108F | CATCAACATCTCAATTCCACCTGGC | |  |
| BHQ10_004108R | GAGCCGATCAAGACGGATCC | |  |
| BHQ10_003783F | CCGACGCTAGGTATCTAAATGACAGATC | |  |
| BHQ10_003783R | CCTGCACCTCGGTTATGTATAAGGAATG | |  |
| BHQ10_004182F | GTCGAGACGCACTTGGTAATTAGC | |  |
| BHQ10_004182R | GAATGAGCCGCTTGCAATAGGC | |  |
