# Supplementary material for: Molecular characterization and transcriptomic analysis of a novel polymycovirus in the fungus Talaromyces amestolkiae
Source: Front Microbiol. 2022 Oct 26;13:1008409. doi: 10.3389/fmicb.2022.1008409 (PMC9645161; doi:10.3389/fmicb.2022.1008409)
Supplement: Supplementary file 3 [file Table_2.DOCX]

**Table S2** Polymycoviruses and related viruses.

| **Virus** | **Abbreviation** | **Accession number for conserved proteins** | | | **Ref.** |
| --- | --- | --- | --- | --- | --- |
|  |  | **RdRP** | **MTR** | **PAS rp** |  |
| Alternaria alternata polymycovirus 1 | AaPmV1 | QVK45096 | QVK45098 | - | Ma et al., 2022 |
| Alternaria tenuissima virus | - | AJP08049 | - | - | unpublished |
| Aspergillus fumigatus tetramycovirus 1 | AfuTmV1 | CDP74618 | CDP74620 | CDP74621 | Kanhayuwa et al., 2015 |
| Aspergillus fumigatus polymycovirus 1（J-YC） | AfuPmV1 | BCH36613 | BCH36615 | BCH36616 | Zoll et al., 2018 |
| Aspergillus fumigatus polymycovirus 1（V-181-30） | AfuPmV1 | AXE72937 | AXE72939 | AXE72940 | Zoll et al., 2018 |
| Aspergillus fumigatus polymycovirus 1（MMRC） | AfuPmV1 | BBU42080 | BBU42082 | BBU42084 | Takahashi-Nakaguchi et al., 2020 |
| Aspergillus spelaeus tetramycovirus 1 | AspTmV1 | AYP71805 | AYP71807 | AYP71806 | Nerva et al., 2019a |
| Beauveria bassiana polymycovirus 1 | BbPmV1 | CUS18595 | CUS18597 | CUS18598 | Kotta-Loizou and Coutts, 2017 |
| Beauveria bassiana polymycovirus 2 | BbPmV2 | CUS18599 | ^-^ | - | Kotta-Loizou and Coutts, 2017 |
| Beauveria bassiana polymycovirus 3 | BbPmV3 | CAD7829823 | CAD7829825 | CAD7829826 | Filippou et al., 2021 |
| Beauveria bassiana polymycovirus 4 | BbPmV4 | QRF54813 | QRF54815 | - | Kang et al., 2021 |
| Botryosphaeria dothidea RNA virus 1（YZN115） | BdRV1 | AKE49495 | AKE49497 | AKE49498 | Zhai et al., 2016 |
| Botryosphaeria dothidea RNA virus 1（XA-3） | BdRV1 | ALZ41794 | ALZ41796 | - | unpublished |
| Cladosporium cladosporioides virus 1 | CcV1 | AII80567 | AII80569 | AII80570 | unpublished |
| Colletotrichum camelliae filamentous virus 1 | CcFV1 | ASV63092 | ASV63094 | ASV63095 | Jia et al., 2017 |
| Fusarium redolens polymycovirus 1 | FrPmV1 | QDH44656 | QDH44658 | QDH44659 | Mahillon et al., 2019 |
| Hadaka virus 1（1NL） | HadV1 | BCM78286 | BCM78288 | - | Sato et al., 2020b |
| Hadaka virus 1（7n） | HadV1 | BBU94038 | BBU94040 | - | Sato et al., 2020b |
| Magnaporthe oryzae polymycovirus 1 | MoPmV1 | QAU09249 | QAU09251 | QAU09252 | unpublished |
| Mycovirus M7 | - | QLF97276 | - | - | García et al., 2020 |
| Penicillium brevicompactum tetramycovirus 1 | PbTmV1 | AYP71801 | AYP71803 | AYP71802 | Nerva et al., 2019a |
| Penicillium digitatum polymycovirus 1 | PdPmV1 | AVZ65983 | AVZ65985 | AVZ65986 | Niu et al., 2018 |
| Penicillium janthinellum polymycovirus 1 | PjPmV1 | BCJ03666 | BCJ03668 | BCJ03670 | Sato et al., 2020a |
| Phaeoacremonium minimum tetramycovirus 1 | PmTMV1 | QDB74985 | QDB74987 | QDB74988 | Nerva et al., 2019b |
| Plasmopara viticola lesion associated polymycovirus 1 | PvaPolymyco1 | QHG11067 | QHG11068 | - | Chiapello et al., 2020 |
| Plasmopara viticola lesion associated polymycovirus 2 | PvaPolymyco2 | QHG11070 | QHG11069 | QHG11071 | Chiapello et al., 2020 |
| Plasmopara viticola lesion associated polymycovirus 3 | PvaPolymyco3 | QHG11072 | - | - | Chiapello et al., 2020 |
| Plasmopara viticola lesion associated polymycovirus 4 | PvaPolymyco4 | QHG11073 | - | - | Chiapello et al., 2020 |
| Plasmopara viticola lesion associated polymycovirus 5 | PvaPolymyco5 | QHG11074 | - | QHG11076 | Chiapello et al., 2020 |
| Sclerotinia sclerotiorum tetramycovirus 1 | SstRV1 | AWY10945 | AWY10947 | - | Mu et al., 2017 |

-: no Genbank accession number
