# Supplementary material for: Molecular characterization and transcriptomic analysis of a novel polymycovirus in the fungus Talaromyces amestolkiae
Source: Front Microbiol. 2022 Oct 26;13:1008409. doi: 10.3389/fmicb.2022.1008409 (PMC9645161; doi:10.3389/fmicb.2022.1008409)
Supplement: Supplementary file 5 [file Table_5.DOCX]

**Table S6** Metabolism related genes

| ID | Function annotation |
| --- | --- |
| BHQ10_002586 | Sterol regulatory element-binding protein |
| BHQ10_009385 | Amino oxidase |
| BHQ10_004513 | Pleiotropic drug resistance |
| BHQ10_006896 | Polyprenyl synthetase |
| BHQ10_004280 | Cytochrome P450 |
| BHQ10_007724 | Major receptor superfamily |
| BHQ10_007059 | Sugar isomerase |
| BHQ10_008420 | Fungal hydrophobin |
| BHQ10_005051 | AMPbinding enzyme |
| BHQ10_004108 | ATP binging cassette |
| BHQ10_003783 | Prenyltransferases |
| BHQ10_004182 | Flavoprotein |
