# Supplementary material for: Molecular characterization and transcriptomic analysis of a novel polymycovirus in the fungus Talaromyces amestolkiae
Source: Front Microbiol. 2022 Oct 26;13:1008409. doi: 10.3389/fmicb.2022.1008409 (PMC9645161; doi:10.3389/fmicb.2022.1008409)
Supplement: Supplementary file 6 [file Data_Sheet_2.docx]

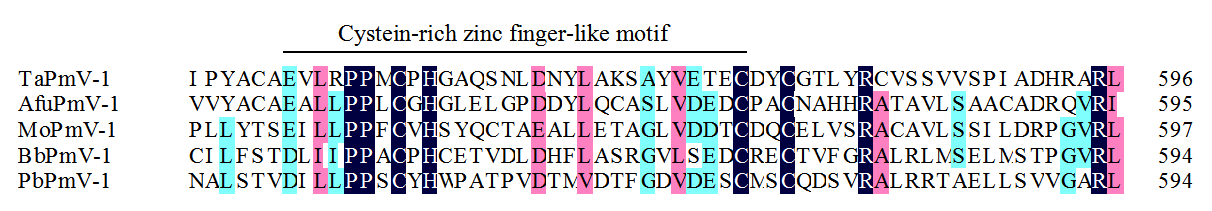


**Figure S1** Multiple sequence alignment of the cysteine-rich zinc finger-like motif identified in the hypothetical proteins encoded by dsRNA2 of TaPmV-1and other polymycoviruses.


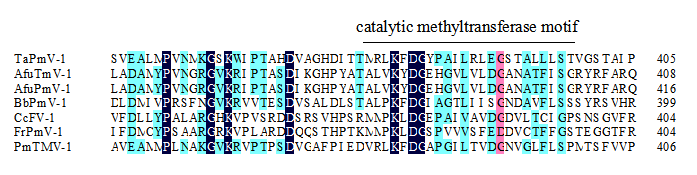


**Figure S2** Multiple sequence alignment of the catalytic MTR motif identified in the proteins encoded by dsRNA3 of TaPmV-1 and other polymycoviruses.


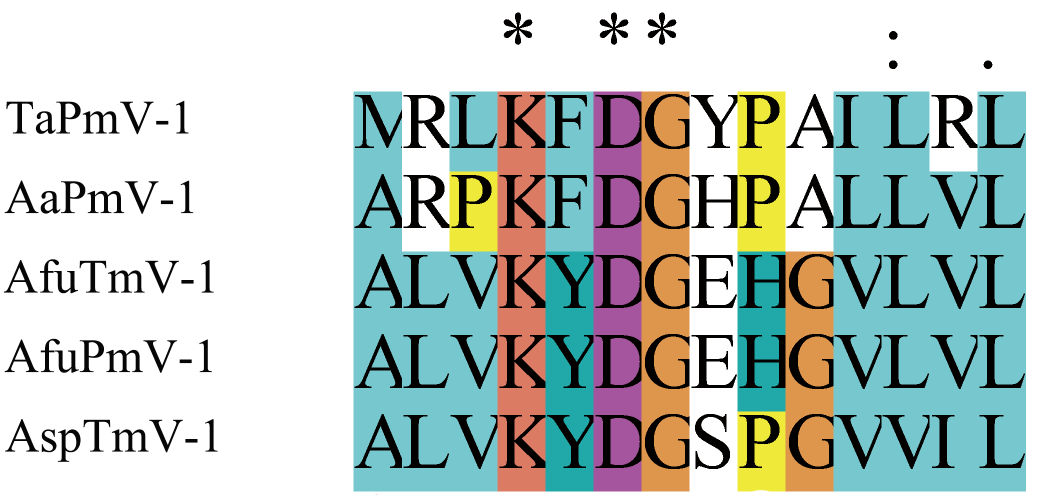


**Figure S3** Multiple sequence alignment of the regions of proteins putatively encoded by TaPmV-1, AaPmV1, AfuTmV-1, AfuPmV-1 and AspTmV-1 that mediate the in 5’- capping of RNA.


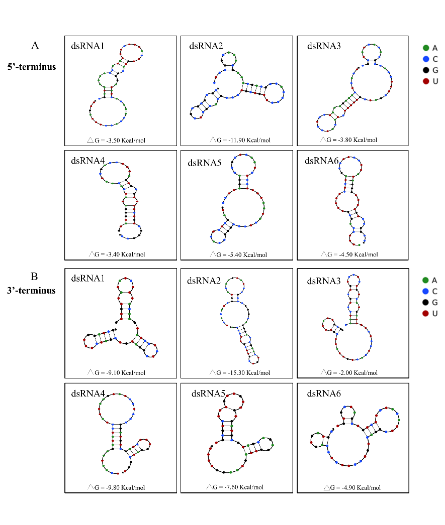


**Figure S4** Predicted secondary structures of 5’-UTRs and 3’-UTRs of dsRNAs 1-6 of TaPmV-1. (A) Secondary structures of the 5’-UTRs of dsRNA1-dsRNA6. (B) Secondary structures of the 3’-UTRs of dsRNA1- dsRNA6.


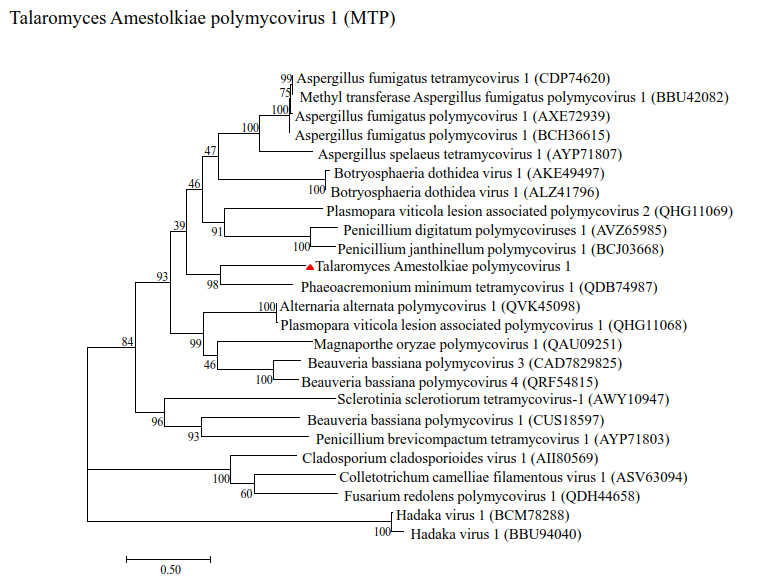


**Figure S5** Phylogenetic analyses of TaPmV-1 and selected polymycoviruses. Phylogenetic analyses of TaPmV-l based on the sequences of MTP.


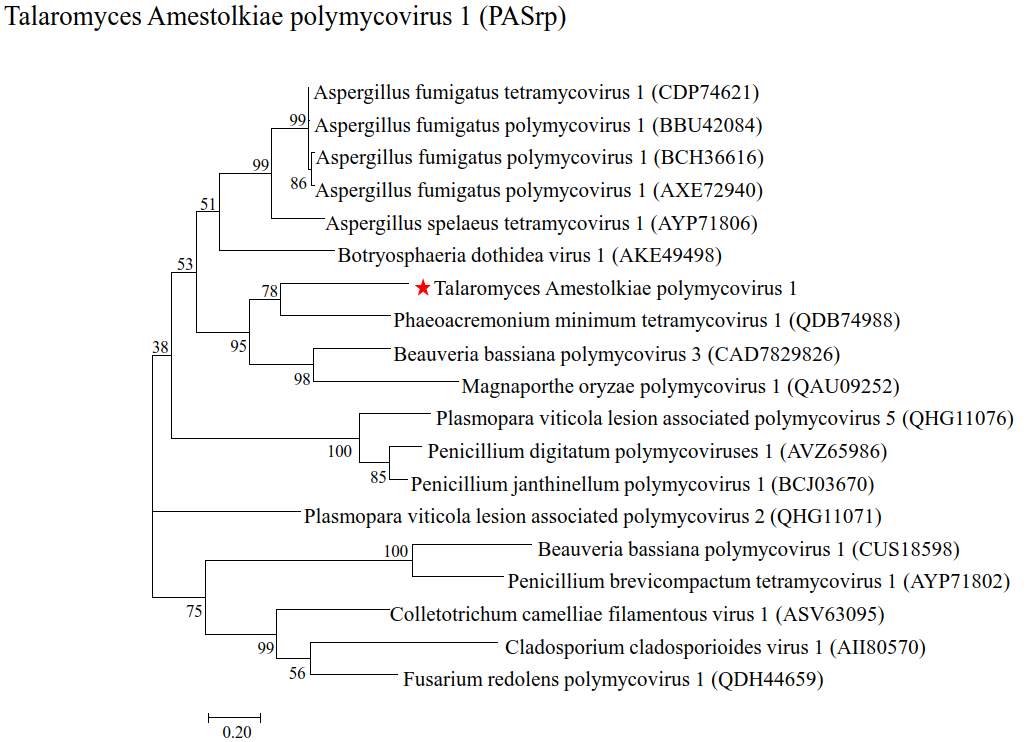


**Figure S6** Phylogenetic analyses of TaPmV-1 and selected polymycoviruses. Phylogenetic analyses of TaPmV-l based on the sequences of PASrp.


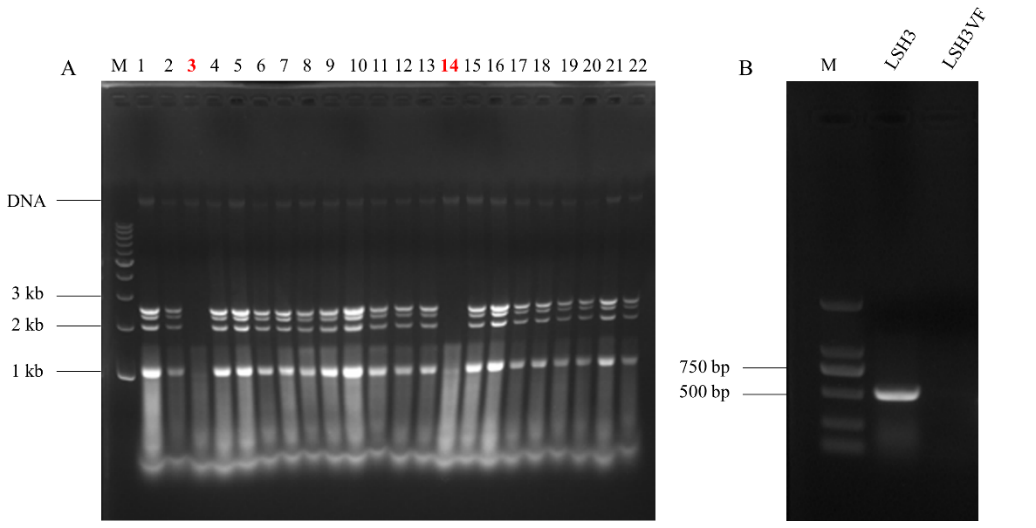


**Figure S7** Detection of TaPmV-1 in *T. amestolkiae*. (A) Detection of TaPmV-1 in *T. amestolkiae* following treatment with the protoplast preparation and regeneration approach, using CF11 cellulose columns for extraction followed by gel electrophoresis. (B) The presence of the TaPmV-1 genome in the strain LSH3 and the absence of the TaPmV-1 genome in the strain LSH3VF were confirmed by RT-PCR.
